# Sexual Display and Mate Choice in an Energetically Costly Environment

**DOI:** 10.1371/journal.pone.0015279

**Published:** 2010-12-09

**Authors:** Megan L. Head, Bob B. M. Wong, Robert Brooks

**Affiliations:** 1 School of Biological, Earth and Environmental Sciences, The University of New South Wales, Sydney, New South Wales, Australia; 2 Evolution, Ecology and Genetics, Research School of Biology, The Australian National University, Canberra, Australian Capital Territory, Australia; University of Jyväskylä, Finland

## Abstract

Sexual displays and mate choice often take place under the same set of environmental conditions and, as a consequence, may be exposed to the same set of environmental constraints. Surprisingly, however, very few studies consider the effects of environmental costs on sexual displays and mate choice simultaneously. We conducted an experiment, manipulating water flow in large flume tanks, to examine how an energetically costly environment might affect the sexual display and mate choice behavior of male and female guppies, *Poecilia reticulata*. We found that male guppies performed fewer sexual displays and became less choosy, with respect to female size, in the presence of a water current compared to those tested in still water. In contrast to males, female responsive to male displays did not differ between the water current treatments and females exhibited no mate preferences with respect to male size or coloration in either treatment. The results of our study underscore the importance of considering the simultaneous effects of environmental costs on the sexual behaviors of both sexes.

## Introduction

Costs associated both with expressing sexual traits and with choosing mates based on these traits are of central importance to understanding the evolutionary potential of sexual selection. Handicap models of sexual selection, for example, show that sexual ornaments can be reliable indices of genetic quality (sensu [1]) if they are condition-dependent and costly to bear [2]. Empirical evidence also suggests that the expression of such traits may be highly sensitive to environmental costs (reviewed in [3]). The presence of potential predators, for instance, has been shown to reduce sexual displays in a range of species, including fiddler crabs (*Uca beebei* – [4]), pipefishes (*Syngnathus typhle* – [5]), and frogs (*Physalaemus pustulosus* – [6]).

Relative to the costs of display, the costs of mate choice (by both males *and* females) have received little empirical attention. However, costs of mate choice are also important in determining the outcome of sexual selection [7]. If the benefits of mate choice remain constant, females are predicted to become less choosy as the costs of choice increase [7], [8]. Thus, the costs of mate choice may influence the types of sexual traits that are chosen and the benefits that are gained by mating with individuals with chosen traits. As is found for sexual display, there is evidence that mate choice may also be influenced by environmental factors, such as predation risk [9]–[11], energetic costs [12],[13], food availability [14]–[16], and parasitic infection [17],[18].

Although it is evident that environmental factors have important influences on the evolution of both sexual display and mate choice, few studies have manipulated environmental costs for both simultaneously (but see e.g. [19]–[21]). Environmental factors that differ between habitats, such as food availability, predation risk, or parasitic infection are unlikely to affect one sex without affecting the other. This is particularly true when the behavior of members of one sex also influences that of potential suitors. For example, in the presence of predators, male guppies, *Poecilia reticulata*, alter their mating behavior in response to altered female behavior rather than in response to the predator itself [22]. To obtain a more complete understanding of selection acting on male and female mating behavior in different environments, it is necessary to simultaneously manipulate the costs of both sexual display and mate choice.

In this study we examined the effects of water current on male reproductive behavior, and both male and female mate choice in guppies. Previous studies on guppies have shown that females prefer males with high display rates and bright coloration (reviewed in [23]), while males prefer large females [24],[25]. Since swimming against a current is energetically demanding [26], we predicted that an increase in water current would lead to 1) a general decline in costly sexual displays and 2) a decrease in the choosiness of both males and females (because the costs of choice would be greater than when there was no current).

## Materials and Methods

Guppies used in this experiment were fourth generation laboratory stock, descended from 500 wild-caught individuals from Alligator creek, near Townsville, Australia. The water currents of creeks in this region vary both spatially and temporally, ranging between 0.003–0.483 m s^−1^. Guppies within these creeks are able to move between areas of differing currents (pers. obs.), and in other populations it has been shown that males and females are differentially distributed within streams with regard to water velocity [27],[28]. The maximum velocity in which males have been observed displaying in wild populations, however, is 0.13 m s^−1^ [28]. Female test subjects were taken from a tank containing only virgins, which had been separated from males upon sexing at 30–40 days. Males came from mixed stock tanks and, thus, had prior sexual experience.

### Experimental protocol

**Figure 1:**
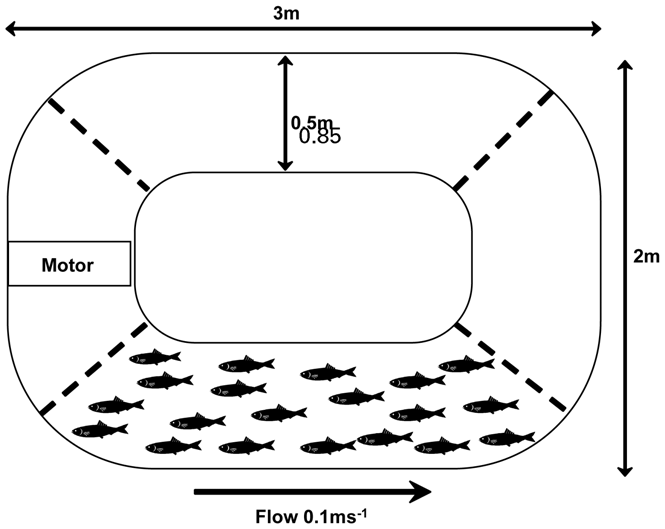
Design of experimental tanks. Fish were confined to one side of the tank, to aid behavioral observations, using mesh barriers (represented by dashed lines).

To examine the effects of water current on both male and female sexual behavior, we set up two treatments: one treatment had no water current (N = 4) and the other had a current of 0.1 m s^−1^ (N = 4). We ran one replicate of each treatment simultaneously using two separate flume tanks (Figure 1), resulting in temporally paired replicates. For each replicate, we introduced ten males and ten females into a flume tank. An outboard motor was secured at one end of each flume and was activated in the water current treatment to provide the desired flow. Guppies were confined to one side of the tank (away from the motor) using black plastic mesh barriers (mesh aperture = 1 mm). The depth of the water was 55 cm. The tank substrate consisted of stones ranging in size from 2–5 cm diameter. This substrate allowed guppies to take refuge from the current (i.e. in eddies) to rest. However, to feed and interact with other individuals, the guppies had to swim in the current. Test subjects were fed once a day with frozen brine shrimp (∼50 ml per tank).

Female guppies were injected with visible red implant elastomer tagging (obtained from Northwest Marine Technology Inc., USA) two weeks before the experiment to allow them to be individually identified during trials. The elastomer was injected into the females' tail muscle using a syringe. We injected the elastomer into one or two different positions (out of six possible positions), so that each female in a tank had a unique marking. Male guppies were individually identified from their distinct color patterns.

All fish were individually weighed before being randomly assigned to their respective treatments. At this stage, we also took an image of the right side of each male using a digital camera. To do so, we first anaesthetized the males by dipping each individual into a slurry of ice for a few seconds [29]. Each male was then laid flat on a piece of white waterproof paper and photographed. A ruler placed next to the guppy was used for calibration. We used Measuremaster 3.44 (Leading Edge Pty Ltd Adelaide, Australia) to measure body and tail area and the area of each color spot from the photograph. From these measures, we calculated the proportion of body area covered by black, orange and iridescent spots.

Guppies were acclimated for one day in the flume tanks, with behavioral observations taking place on the two mornings after acclimation. On each day, focal observations involved watching each male and female for two, three minute periods [23]. Observations alternated between males and females but were otherwise in random order. Following this, we conducted further observations for 20 minutes, where we scanned the tank watching for male displays [14]. When a display was observed, we recorded the identity of the male and female involved, and recorded the females' response to the display (see below). During behavioral trials, light was provided by two reading lamps (60W daylight incandescent bulbs) suspended 30 cm diagonally above each tank.

During focal observations we recorded both male and female sexual behavior. We recorded male reproductive behaviors by tallying the number of times he followed, chased, jockeyed (i.e. two or more males chasing a female), and nipped (i.e. biting the females gonopore region) females. Additionally, we noted the number of times the male engaged in sneak copulations (i.e. approaching the female from behind and inserting the gonopodium in the females' gonopore with no prior display), and performed sigmoid displays (hereafter referred to as the males' “sexual display” since this is a display behaviour used in courtship, where the male shows off his colorful flanks to the female [30]). We also recorded whether a female responded positively to sigmoid displays (i.e. whether the female ceased her current activity and glided towards the male [23]) or not. Female responsiveness was measured as the mean proportion of displays from all males that a female responded to positively [31]. Male attractiveness was measured as the mean proportion of displays (that a male performed to all females) that received a positive response [32]. Female attractiveness was determined as the number of displays that males directed toward her. All work was conducted in accordance with Australian animal ethics guidelines and was covered by The University of New South Wales animal ethics approval number ACEC 01/108.

### Statistical analysis

To test the effects of water current on male behavior we used a generalized linear mixture model with a Tweedie distribution and a log link function, nesting males within replicates. The Tweedie distribution is an appropriate distribution for data that are zero inflated [33]. Significant results were adjusted for false discovery rate [34].

To examine the effects of male coloration and size on female responsiveness as well as female size on male sexual display, we estimated the within-tank regression coefficients. Both the number of displays and the proportion of displays were log transformed, due to non normality, prior to regression analysis. We then paired the coefficients for the tanks according to when they were set up and performed paired *t*-tests on these coefficients to compare the differences in these relationships with respect to treatment, and when differences were present, we performed one sample t-tests to determine if the slopes of the preferences for each treatment were different from zero. All tests were two-tailed.

## Results

### Male behavior

When a water current was present, males displayed less often (Table 1). No other male behaviors showed significant differences between water current treatments (Table 1). Male mate preferences, however, did differ between the treatments (paired t-test: *t* = 3.299, d.f. = 3, p = 0.046). Males were choosier when there was no water current, displaying more often to large females (one sample t-test: mean β ± S.E. = 0.229±0.107, *t* = 3.315, d.f. = 3, p = 0.046). When a current was present, however, this relationship between male display rate and female size disappeared (one sample t-test: mean β ± S.E. = −0.058±0.158, t = 0.682, d.f. = 3, p = 0.544).

**Table 1:** The effect of treatment on the number of male reproductive behaviors. Mean behaviors are reported per male per minute.

	Mean ± S.E.			
Response variable	No current	Current	*χ^2^*	p
Sexual display	0.496±0.067	0.263±0.116	8.328	0.004*
Sneak copulation	0.275±0.062	0.396±0.152	0.194	0.660
Follow	0.229±0.049	0.179±0.071	2.173	0.140
Chase	0.088±0.028	0.075±0.038	0.459	0.498
Nip	0.050±0.018	0.054±0.036	0.504	0.478
Jockey	0.050±0.034	0.125±0.057	0.430	0.512

*remains significant after adjustment for false discovery rate p_FDR_  = 0.024.

### Female behavior

There were no differences in female responsiveness (mean ± S.E.; no current  = 0.111±0.032, current = 0.210±0.041; GLMM, *F* = 0.791, p = 0.374) between treatments. Female choosiness also did not differ between our treatments with regards to male phenotype (Table 2) or female weight (mean β ± SE, no current  = −0.023±0.016, current  = 0.021±0.023, t = −1.176, d.f. = 3, p = 0.162).

**Table 2:** Comparison of female preferences between the no current and current treatments.

	Mean β±S.E.				
	No current	Current	*t*	d.f.	P
Weight	0.014±0.036	0.024±0.026	−0.882	3	0.444
Tail area	0.007±0.029	0.098±0.105	−1.060	3	0.368
Black area	0.019±0.067	−0.006±0.009	0.389	3	0.724
Orange area	−0.002±0.029	0.035±0.039	−0.655	3	0.560
Iridescent area	−0.008±0.023	−0.026±0.045	0.318	3	0.770

Female preferences are expressed as the relationship between the proportion of male displays receiving a positive response and male phenotype. Color areas are expressed as the proportion of male body area covered.

## Discussion

Previous studies have shown that swimming against a current can be an energetically demanding activity [26]. As a consequence, individuals in faster water currents should have less energy available to invest in sexual display and mate choice [12]. Consistent with this prediction, we observed a decline in sexual display and mate choice in our water current treatment for males. However, female behaviour remained the same in both treatments.

Male guppies responded to an energetically costly environment by reducing the frequency of sexual displays to females. Magellan & Magurran [28] also show that males perform fewer sexual displays in greater water velocities. A reduction in display activity has also been reported in guppies that were either food-limited (e.g. [35]) or exposed to heavy parasite loads [36]. Hence, in guppies, there appears to be an important nexus between energetic state and the expression of male sexual displays. Our findings are also concordant with studies in other taxa. For instance, in wolf spiders *Hygrolycosa rubrofasciata*, food-limited males reduced the rate of their drumming displays [37], and in crickets *Teleogryllus commodus*, males subjected to lower quality diets also called less frequently than those offered a diet rich in protein [1].

In the presence of a water current, male guppies were also less discerning compared to those tested in still water. Specifically, when there was no water current, males displayed more often to large females but no such preference was observed in the water current treatment. A preference for larger females has been reported in a range of species, including guppies [24],[25]. Such a preference is likely due to a positive size-fecundity relationship, which means larger females are often more valuable in terms of reproductive value [25],[38]. A lack of preference for large females in the water current treatment suggests, however, that males may be capable of adjusting courtship behavior in response to prevailing environmental conditions. Due to the increased energetic costs of swimming in a current, searching for mates is likely to be more costly in the presence of a water current. In this regard, our results are consistent with models of mate choice evolution predicting that choosiness should diminish when sampling costs are high [39],[40]. Alternatively, since the ability to swim in a current may be related to body size [27],[41], larger females could simply be more adept at avoiding courting males and this, in turn, might explain the lack of male preference for large females in the water current treatment. Intriguingly, several studies have shown that females will deliberately swim into water currents to avoid unwanted courtship attempts [28],[42].

Females on the other hand showed no significant differences in behaviour between the still and flowing water treatments. This is despite expected costs associated with living in a water current [12],[26], and evidence from food manipulation studies in this population of guppies that have shown that female responsiveness is condition-dependent [14]. That male reproductive behaviour differs between water current treatments while female behaviour does not, suggests that female behaviour may be less sensitive to the costs that living in a water current imposes on mate choice than males are.

The fact that we found no relationship between male sexual traits and male attractiveness in either of our treatments is unexpected: females from this population have previously been shown to choose males based on their coloration [14],[29]. In this regard, we do not rule out the possibility of low statistical power in explaining the lack of relationship in the current study. However, another possible explanation is that, in contrast to most laboratory studies on guppies, fish in our experiment were kept at relatively low densities (0.03 fish/litre) to more accurately mimic wild populations. It has been suggested that, at low population densities, mate choice may decrease due to low encounter rates with potential mates [43]. Such a possibility warrants further examination.

The evolutionary consequences of differences in male and female mating behavior, as a result of cost-related differences in water currents, are likely to depend on the scale of environmental variation that exists in the field. Small-scale differences in water velocity within populations could, on the one hand, favour different phenotypes under different conditions [44],[45],[46], thus maintaining variation in these traits [47],[14]. On the other hand, large scale geographic differences in water velocity may lead to population divergence of traits which could, in turn, facilitate reproductive isolation and speciation [48],[49]. In light of these considerations, future studies may wish to examine differences in the response of both sexes to environmental costs within, as well as between, populations.

## References

[1] Hunt J, Brooks R, Jennions MD, Smith MJ, Bentsen CL (2004). High-quality male field crickets invest heavily in sexual display but die young.. Nature.

[2] Zahavi A (1975). Mate selection - A selection for a handicap.. J Theor Biol.

[3] Kotiaho JS (2001). Costs of sexual traits: a mismatch between theoretical considerations and empirical evidence.. Biol Rev.

[4] Koga T, Backwell PRY, Jennions MD, Christy JH (1998). Elevated predation risk changes mating behavior and courtship in a fiddler crab.. Proc R Soc B.

[5] Fuller R, Berglund A (1996). Behavioral responses of a sex-role reversed pipefish to a gradient of perceived predation risk.. Behav Ecol.

[6] Tuttle MD, Taft LK, Ryan MJ (1982). Evasive behavior of a frog in response to bat predation.. Anim Behav.

[7] Jennions MD, Petrie M (1997). Variation in mate choice and mating preferences - a review of causes and consequences.. Biol Rev.

[8] Kokko H, Brooks R, McNamara JM, Houston AI (2002). The sexual selection continuum.. Proc R Soc B.

[9] Stoner G, Breden F (1988). Phenotypic differentiation in female preference related to geographic variation in male predation risk in the Trinidad guppy (*Poecilia reticulata*).. Behav Ecol Sociobiol.

[10] Forsgren E (1992). Predation risk affects mate choice in a gobiid fish.. Am Nat.

[11] Booksmythe I, Detto T, Backwell PRY (2008). Female fiddler crabs settle for less: the travel costs of mate choice.. Anim Behav.

[12] Milinski M, Bakker TCM (1992). Costs influence sequential mate choice in sticklebacks, *Gasterosteus aculeatus.*. Proc R Soc B.

[13] Wong BBM, Jennions MD (2003). Costs influence male mate choice in a freshwater fish.. Biol Lett.

[14] Syriatowicz A, Brooks R (2004). Sexual responsiveness is condition-dependent in female guppies, but preference functions are not.. BMC Ecology.

[15] Hunt J, Brooks R, Jennions MD (2005). Female mate choice as a condition-dependent life-history trait.. Am Nat.

[16] Hebets EA, Wesson J, Shamble PS (2008). Diet influences mate choice selectivity in adult female wolf spiders.. Anim Behav.

[17] Poulin R (1994). Mate choice decisions by parasitized female upland bullies, *Gobiomorphus breviceps.*. Proc R Soc B.

[18] López S (1999). Parasitized female guppies do not prefer showy males.. Anim Behav.

[19] Sih A, Krupa J, Travers S (1990). An experimental study on the effects of predation risk and feeding regime on the mating behavior of the water strider.. Am Nat.

[20] Grether GF, Kolluru GR, Rodd FH, de la Cerda J, Shimazaki K (2005). Carotenoid availability affects the development of colour-based mate preference and the sensory bias to which it is genetically linked.. Proc R Soc B.

[21] Dunn AM, Dick JTA, Hatcher MJ (2008). The less amorous Gammarus: predation risk affects mating decisions in *Gammarus duebeni* (Amphipoda).. Anim Behav.

[22] Evans JP, Kelley JL, Ramnarine IW, Pilastro A (2002). Female behavior mediates male courtship under predation risk in the guppy (*Poecilia reticulata*).. Behav Ecol Sociobiol.

[23] Houde AE (1997). Sex, color, and mate choice in guppies..

[24] Dosen LD, Montgomerie R (2004). Female size influences mate preferences of male guppies.. Ethology.

[25] Herdman EJE, Kelly CD, Godin JGJ (2004). Male mate choice in the guppy (*Poecilia reticulata*): Do males prefer larger females as mates?. Ethology.

[26] Standen EM, Hinch SG, Healey MC, Farrell AP (2002). Energetic costs of migration through the Fraser river canyon, Brithish Columbia, in adult pink (*Oncorhynchus gorbuscha*) and sockeye (*Onchorhynchus nerka*) salmon as assessed by EMG telemetry.. Can J Fish Aquat Sci.

[27] Karino K, Orita K, Sato A (2006). Long tails affect swimming performance and habitat choice in the male guppy.. Zool Sci.

[28] Magellan K, Magurran AE (2006). Habitat use mediates the conflict of interest between the sexes.. Anim Behav.

[29] Gamble S, Lindholm AK, Endler JA, Brooks R (2003). Environmental variation and the maintenance of polymorphism: the effect of ambient light spectrum on mating behavior and sexual selection in guppies.. Ecol Lett.

[30] Magurran AE, Seghers BH (1994). A cost of sexual harassment in the guppy, *Poecilia reticulate.*. Proc R Soc B.

[31] Endler JA, Houde AE (1995). Geographic variation in female preferences for male traits in *Poecilia reticulate.*. Evolution.

[32] Brooks R, Endler JA (2001). Direct and indirect sexual selection and quantitative genetics of male traits in guppies (*Poecilia reticulata*).. Evolution.

[33] Swan T (2006). Generalized estimating equations when the response variable has a Tweedie distribution: An application for multi-site rainfall modelling..

[34] Benjamini Y, Hochberg Y (1995). Controlling the false discovery rate - a practical and powerful approach to multiple testing.. Journal of the Royal Statistical Society Series B-Methodological..

[35] Kolluru GR, Grether GF (2005). The effects of resource availability on alternative mating tactics in guppies (*Poecilia reticulata*).. Behav Ecol.

[36] Houde AE, Torio AJ (1992). Effect of parasitic infection on male color pattern and female choice in guppies.. Behav Ecol.

[37] Kotiaho JS (2000). Testing the assumptions of conditional handicap theory: costs and condition dependence of a sexually selected trait.. Behav Ecol Sociobiol.

[38] Katvala M, Kaitala A (2001). Male choice for current female fecundity in a polyandrous egg-carrying bug.. Anim Behav.

[39] Real L (1990). Search theory and mate choice 1. Models of single-sex discrimination.. Am Nat.

[40] Crowley PH, Travers SE, Linton MC, Cohn SL, Sih A (1991). Mate density, predation risk, and the seasonal sequence of mate choices: A dynamic game.. Am Nat.

[41] Ghalambor CK, Reznick DN, Walker JA (2004). Constraints on adaptive evolution: The functional trade-off between reproduction and fast start swimming performance in the Trinidadian guppy (*Poecilia reticulate*).. Am Nat.

[42] Kodric-Brown A, Nicoletto PF (2005). Courtship behaviour, swimming performance, and microhabitat use of Trinidadian guppies.. Env Biol Fish.

[43] Eshel I (1979). Sexual selection, population density, and availability of mates.. Theor Pop Biol.

[44] Nicoletto PF (1996). The influence of water velocity on the display behavior of male guppies, *Poecilia reticulate.*. Behav Ecol.

[45] Nicoletto PF, Kodric-Brown A (1999). The relationship among swimming performance, courtship behavior, and carotenoid pigmentation of guppies in four rivers of Trinidad.. Env Biol Fish.

[46] Langerhans RB (2008). Predictability of phenotypic differentiation across flow regimes in fishes.. Integr Comp Biol.

[47] Brooks R (2002). Variation in female mate choice within guppy populations: population divergence, multiple ornaments and the maintenance of polymorphism.. Genetica.

[48] Lande R (1981). Models of speciation by sexual selection on polygenic traits.. Proc Natl Acad Sci USA.

[49] Boughman JW (2001). Divergent sexual selection enhances reproductive isolation in sticklebacks.. Nature.

